# Unchartered Territory: Understanding Public Compliance During the Covid-19 Pandemic and the Effectiveness of UK Government Communications

**DOI:** 10.5964/ejop.13965

**Published:** 2025-08-29

**Authors:** Ashley Cartwright, Jason Roach, Liam Scott Curran

**Affiliations:** 1 Crime and Policing Research Centre, University of Huddersfield, Huddersfield, United Kingdom; University of South Wales, Cardiff, United Kingdom

**Keywords:** compliance, social distancing, COVID 19, behavioural change

## Abstract

The COVID-19 pandemic presented unpresented challenges to societies and the way in which we live, everyday behaviours became prohibited and various parts of the economy were completely shut down in the United Kingdom. Such draconian and radical changes to everyday life are indeed important to study and the present paper captures a representative response of the compliance to social distancing measures implemented in the United Kingdom. This paper explores the effectiveness of the UK Government’s messaging aimed at inducing conformity, whilst considering alternative experimental messages designed to influence conformity by targeting demographics using the principles of NUDGE theory (Thaler & Sunstein, 2008). An online cross-sectional survey was administered in May/June 2020 to 1028 residents of the UK. A cluster analysis was performed to identify different demographic profiles associated with rule breaking. The findings of the present paper outlined clearly that the UK public struggled to adhere to the social distancing rules implemented with relatively low rates of complete compliance and identified two groups of individuals who although only represent a small percentage of the sample (< 20%) were accountable for the majority of rule breaking behaviour. The findings provide an indication of which measures were viewed as more serious and as such less likely to broken, alongside which demographic groups were the least compliant. Regarding the Government’s messaging used during the pandemic, this was demonstrated to be more effective than the hypothetical communications used in the present study. The present paper offers, important insights into specific aspects of non-conformity, including contact with the Police during this time. The present paper offers important learning lessons to improve adherence to social distancing in the future by providing a breakdown of the areas where social distancing is most likely to breached and an indication of who is likely to do this.

The impact of the COVID-19 pandemic was felt, albeit to differing degrees, the world-over. In the UK the pandemic led to a significant disruption to health, wellbeing, and the economy, on a scale and in ways not experienced by society for centuries. Throughout the duration of the pandemic, in the UK it was consistently stated by many scientists and members of the Government, that the best defence against being totally overwhelmed by COVID-19, particularly the National Health Service (NHS), necessitated a significant behaviour change by the UK population, to slow the transmission of the virus. Understanding public compliance with rules that prohibit previously natural behaviours, such as visiting public spaces and socialising with friends and family from different households, is an opportunity to be seized. This will allow us to learn important lessons for dealing with future pandemics or other events requiring rapid and unnatural behaviour changes by large populations.

To date, there have been few published research studies which have explored conformity or public compliance with rules of social distancing, but these have come to broadly similar conclusions as will be outlined within our literature review. So how did the UK Government attempt to achieve a high-level of public compliance with the Covid-19 guidance and rules during the pandemic? To encourage the necessary high-level of public compliance with Covid-19 rules (e.g., social distancing), the UK Government took advice recommended by the UK Behavioural insights Team (Yates, 2020) known colloquially at least as the ‘Nudge Unit’ as they utilised heavily the ‘Nudge’ approach to behavioural change, pioneered by Thaler and Sunstein (2008). To date, there have been very few studies examining nudging compliance with lockdown measures or evaluating communications for influencing compliance.

The present paper is not an endorsement of nudge theory but more an evaluation of the approach taken to influence compliance during the COVID 19 pandemic in the United Kingdom. Indeed, even if one accepts the theoretical basis for nudges, the way in which messages were delivered by the UK Government is argued here to be problematic. The reason for this is that the messaging delivered by the UK Government were addressed to the nation without knowing the discernible demographic differences aimed at influencing conformity.

The present paper takes an exploratory approach to look back at the COVID-19 pandemic in the UK and learn lessons for future pandemics about what may work in influencing compliance. To do this, it is argued here, that first we need to understand the UK public’s compliance with Covid-19 social distancing rules to a greater extent than is currently known and determine ‘who’ needs to be influenced and ‘what’ behaviours need to be influenced. Secondly, an evaluation of the Government’s messaging for compliance needs to be retrospectively evaluated alongside alternative communications that were not utilised during the pandemic. To our knowledge, this has not been done before and the present paper, therefore, breaks new exploratory ground.

## Literature Review

To set the appropriate context, we begin with a short review of what ‘is already known’, about degree of compliance by the UK population to the social distancing rules introduced by the UK Government in response to the Covid-19 pandemic. Smith et al. (2020) used data collected from a representative sample of the UK population, to produce a cross-sectional survey of UK public compliance with social distancing rules. They found that although only 9.7% of the sample reported as having COVID-19 symptomology within their household 75.1% of these reported that they had still left their homes within the previous 24 hours, therefore breaching arguably the most important self-isolation (self-quarantine) rule thereby increasing the chances of the virus being transmitted to others (Smith et al., 2020). In terms of the demographics of the rule breachers, rather unsurprisingly, the males in this group were found to be more likely to be ‘non-compliant’ with the rules than their female counterparts. Unsurprising in the sense that centuries of criminological study highlight young males to be less-risk adverse and more likely to break rules and engage in antisocial behaviour (Roach & Pease, 2013). In terms of identifying those factors most associated with non-compliance, the authors found that those reported as being most prominent included, not believing that social distancing measures were effective, low estimations of the severity of the virus (i.e., that they will be okay should they contract Covid-19), and a low estimate of the extent to which others were complying the rules (Smith et al., 2020).

Hills and Eraso (2021) demonstrated further evidence in their study comprising self-report data for 681 residents of North London (UK); again, participants reported that they did not comply strictly with the social distancing rules. Indeed, almost half of them reported that they had ignored UK Government rules and guidance intentionally. Influential factors identified for such participant ‘non-compliance’ included: a perceived lower vulnerability to the virus, having high education attainment levels, and their individual political beliefs. Moreover, only a maximum of 8% of participants in this study self-reported that they had recently complied with all the current social distancing rules during a recent (to the conducting of the research) two-week period.

In a study of public behaviour involving participants from the Republic of Ireland and Northern Ireland, Darker et al. (2021) found that those who reported breaking the current social distancing rules, were more likely to be; male, be members of lower socioeconomic groups, be young, be healthcare workers, reported being low in affect (e.g., low mood), and were less likely to live in households with children. One of the more illuminating insights into the level of compliance by the UK public is documented in the Ganslmeier et al. (2022) study, which involved 105,000 UK participants. The study revealed a clear relationship between warmer weather conditions and a lack of compliance with regulations. It indicated that, while compliance was generally high, it waned as the months became warmer. Non-compliance was particularly associated with young males aged 20 – 30, part-time employees, divorced individuals, and parents of more than two children.

Further UK based research focused on public behaviour during the Covid-19 pandemic by Wu et al. (2022) found that compliance with social distancing tended to decay (i.e., decrease) over time. Measured at three different points in time during the pandemic, they identified that public compliance significantly reduced as reported by participants in a representative UK sample. This is a consistent with other studies in other European countries, (Reinders Folmer et al., 2021; Rosman et al., 2021) and strongly suggests that if public compliance is greatest at the beginning of a pandemic (e.g., during the first lockdown periods) then this is the optimum time for introducing those proven to be the most effective measures to encourage the public to adopt and sustain behavioural changes for longer periods of time. Put simply, although initial compliance is likely to be high, to prevent ‘compliance decay’, then effective messages need to be incorporated if the desired behavioural changes, in terms of social distancing, are to be achieved for longer. This presupposes that we know what the most effective these messages are of course. This is the second purpose of this paper and an area that has not been well explored.

## Theoretical Background

We only offer a brief introduction to the theoretical foundations of the ‘Nudge’ approach and the work of the Behavioural Insights Team to provide the reader with an insight into the theoretical strategy that is reported to have been used by the policy makers. Thaler and Sunstein’s (2008) ‘Nudge approach’ rests on the premise that human behaviour is impressionable through the arrangement and availability of different choices which can and do affect human decision-making. Not an entirely new approach to human decision-making, the foundations for nudge rest firmly with behavioural psychologists such as James Gibson and the pioneering work of Daniel Kahneman and Amos Tversky identifying common cognitive short-cuts and bias in human decision-making, in particular, how the order of presentation of options can influence the choices and decisions that we make. For example, if asked to recall a list of words, we are most likely to remember those at the beginning and those at the end of the list (Roach, 2023). Thaler and Sunstein (2008) state that ‘nudging’ can be used to encourage the adoption of prosocial behaviour, such as increasing the number of willing human organ donors, or compliance with tax demands. In a crime and policing context, more recent explorations of the power of nudge have seen it used to reduce crime, for example by ‘nudging’ potential victims of theft to be a little more safety conscious when leaving their vehicles (e.g., to encourage them to remember lock them’ (Roach et al., 2017).

It is therefore no surprise that the nudge approach has become an attractive means by which policy makers and politicians can influence the decision-making and consequently the behaviour of members of the public. It is generally accepted that to stand a chance of being effective, ‘Nudges’ must be bespoke to those who you wish to influence (Roach, 2023). This means that to influence someone then, you need to know as much about them as possible to resonate and relate to them (Roach, 2023). Nudging therefore works with individuals and groups, not with entire populations made up of many different demographics (e.g., as with the UK) as there are no ‘one-size fits all’ messages which will resonate with all. Likewise, the distributor of the message (i.e., the messenger) needs to resonate with the recipient, or the message will have little impact. As any parent will tell you, if you want to influence your teenage child, then you need a teenage child to deliver the message (Roach, 2023).

Nudge approaches are not without their limitations and have come under criticism in Psychology (Maier et al., 2022) due to the issues associated with evaluating nudges, a lack of clear criteria on the development of nudges and the theoretical framework for developing nudges. Additionally, the effectiveness of nudge interventions in research has been demonstrated to be a concerning issue with some arguing that when biases are corrected for there is very little evidence to support the use of nudges (Maier et al., 2022). The crux of the issue comes down to the fact that there are too many possible behavioural ‘nudges’, too many ways to influence individuals, and too many behaviours that are targeted by nudge interventions. Therefore, what might be classed as a nudge in one paper can be extremely different to the next. These limitations are not taken lightly in this paper and although the paper is situated within a nudge framework, the reason for doing this is simply due to this being the approach reported to have been used by the UK Government to influence conformity.

Indeed, there are more established theories of compliance and influence that could be adopted in place of nudges. For example, Cialdini’s (2021) universal principles of compliance, which have been well-documented for effectively influencing behaviour. Cialdini’s principles suggest the following:

- Reciprocity — We feel compelled to return favours.
- Commitment and Consistency — We prefer to act in accordance with our past behaviour.
- Social Proof — We look to others to determine how to act.
- Authority — We are more likely to comply with those we perceive as credible or knowledgeable.
- Liking — We are more likely to comply with those we like.
- Scarcity — We act urgently when things are available in limited quantities.

Returning to a Covid-19 UK context, there are many theoretical frameworks that could have been utilised during the COVID 19 pandemic to improve compliance. However, NUDGE Theory is simply used here as it is what the UK Government were utilising at the time of the pandemic. To our knowledge the only attempt to evaluate this theory in the context of pandemic compliance comes from a French study by Blayac et al. (2022). This study tested social compliance nudges to determine if they could improve compliance with social distancing interventions. The social compliance nudge involved informing participants about other people’s behaviour and then asking them about their hypothetical future behaviour. Although the study did not demonstrate any significant findings overall, it concluded that the nudge was not effective in a general context. However, the location of the individuals on which the social compliance nudge was based was crucial. As such, to date there is very little evidence to support future pandemics about effective communications strategies for influencing conformity.

## The Present Study

The present study poses two important questions based upon the review of the current literature to provide a better understanding of the UK experience of dealing with the Covid-19 pandemic. Firstly, to what extent did the UK public comply with the rules of social distancing and which rules were adhered to or ignored the most? Secondly, how effective was the UK Government’s use of messages utilising nudge theory in encouraging compliance with those social rules?

## Method

### Design

A cross-sectional online survey was developed and administered via Qualtrics. The survey began collecting participant demographics The second section of the survey was developed to explore what behaviours participants engaged in which were indicative of non-compliance during the COVID pandemic and these items are documented in Table 1.

**Table 1:** Questions Exploring Participant Breaches of the UK Government’s Covid-19 / Guidance

Question	Response options		
Allowed children to socialise with their friends.	Yes	No	NA/prefer not to say.
Visited the shop not for essentials.	Yes	No	NA/prefer not to say.
Met with friends or family not in household.	Yes	No	NA/prefer not to say.
Stockpiled items.	Yes	No	NA/prefer not to say.
Went out in public whilst suffering minor symptoms (did not self-isolate).	Yes	No	NA/prefer not to say.
Drove unnecessarily.	Yes	No	NA/prefer not to say.
Did not ensure I kept 2 meters apart from others.	Yes	No	NA/prefer not to say.
Did not wash hands regularly.	Yes	No	NA/prefer not to say.
Socialised inside a family or friend’s house.	Yes	No	NA/prefer not to say.
Exercised with someone not in household.	Yes	No	NA/prefer not to say.
Used public space not in accordance with rules.	Yes	No	NA/prefer not to say.
Hosted visitors inside own house.	Yes	No	NA/prefer not to say.

The third section of the survey presented participants with several real UK Government messages used, mixed with six ‘made-up messages’ developed specifically to influence different demographics of the UK public, such as young people, people with children, and older people. These are displayed in Table 2. Participants were asked to rate on a ten-point scale (0 = *Not influential* and 10 = *Very influential*) their perceived effectiveness of these communication regarding their future behaviour. Alongside asking participants to judge the effectiveness of these communications participants were also asked to outline the most influential strategy for deploying these communications by indicating who they would listen to the most.

**Table 2:** UK Government Messages and Alternative Demographic Specific Communications to Encourage Compliance With Covid-19 Rules During the Pandemic

Nudge	Communication text	Aim of message and target audience
Government Advert	If you can go out, you can spread it. People will die. Stay home. Protect the NHS. Save Lives.	Government ‘general’ message **—** aimed at all of UK public. Unequivocal **—** ‘stay home’ message used at the beginning of the pandemic 9 March to May 2020).
Government Text	New rules in force now: you must stay at home. More info & exemptions at gov.uk/coronavirus. Stay at home. Protect the NHS. Save Lives.	Continuance of Government ‘general’ message aimed at all of UK public. Unequivocal **—** ‘stay home’ message used in the second period of the pandemic (October to December 2020).
Communication 1 (C1)	You are not just doing it for you. Do it for the ones you love. Do it for those you care about. Do it for everybody.	Aimed at those members of the public with children and extended families.
Communication 2 (C2)	Some will ignore the rules. Some will think that they do not apply to them. Some won’t care about your family and friends. Don’t belong to the some. Belong to the many.	Aimed at young males to encourage them to think about compliance for other’s sake.
Communication 3 (C3)	This is our generation’s war and we will win. But only if we fight it together.	Aimed at older people, trying to tap into a ‘war’ and ‘us and them’ spirit.
Communication 4 (C4)	What’s a few weeks in if it protects the rest of your life?	Targeted at young people and the need to look at the bigger future i.e., their future and whole lives.
Communication 5 (C5)	Remind Mummy and Daddy that to keep you safe, they need to stick to the rules.	Aimed at parents with young children.
Communication 6 (C6)	Although it is tough not seeing your family, it is you they are protecting. They love you. Stay strong for them. You will be together again soon.	Aimed at those members of the public who are isolated or who are potentially vulnerable.

### Sample

To achieve a nationally representative UK population sample, a quota-controlled sampling technique was used, based on age, sex, ethnicity and locality (region). Quotas were calculated using the latest available UK census data (at the time of study) and 1040 participants’ from across the United Kingdom were recruited using an Online Panel, hosted by Panelbase. Panelbase recruited participants offering cash incentives for survey completion. To join Panelbase participants went through a double opt-in process to verify their email addresses. New members provide demographic and lifestyle information to allow researchers to target survey opportunities more effectively. Qualified participants were notified via the membership page and emailed with details of this survey. Participants could choose to complete the survey, and successful completion resulted in a reward deposited into their Panelbase account (the exact amount was not shared with the research team). Data was collected from 28^th^ May 2020 to 18^th^ June 2020. The findings are therefore considered representative of the UK population that use the internet and are representative at a 99% confidence level, with a 4% margin of error based upon the population estimate at the time of data collection (52,673,433) of adults aged 18 years or over living in the United Kingdom. As can be seen in Table 3, 1040 participants took part in the present study and the demographic make-up of our participants matched that of the UK public at the time.

**Table 3:** Participant Demographics

Demographic	*n*	%
Age Variable (*M* = 56.3) (*SD* = 17.6)		
18 – 24	140	13.5%
25 – 34	175	16.8%
35 – 44	163	15.7%
45 – 54	179	17.2%
55 – 64	156	15.0%
65+	227	21.8%
Gender		
Male	508	48.8%
Female	530	51.0%
Other	2	0.2%
Ethnic Group		
White or White British	837	80.5%
BAME	203	19.5%
Region		
Scotland	85	8.2%
Northern Ireland	29	2.8%
Wales	49	4.7%
North East	41	3.9%
North West	114	11.0%
Yorkshire and the Humber	88	8.5%
West Midlands	92	8.8%
East Midlands	75	7.2%
South West	88	8.5%
South East	143	13.8%
East Anglia	97	9.3%
Greater London	139	13.4%
Monthly household income after tax		
£0 – £999	128	12.3%
£1000 – £1999	298	28.7%
£2000 – £3999	393	37.8%
£4000 – £5999	129	12.4%
£6000 – £7999	28	2.7%
£8000 – £9999	10	1.0%
£10,000+	54	5.2%
Type of residential location		
Urban	358	34.4
Rural	209	20.1
Suburban	473	45.5
Living circumstances		
Live alone	176	16.9%
Live with one friend or more	25	2.4%
Live with partner	326	31.3%
Live with partner and children	314	30.2%
Live with children	57	5.5%
Live with parents and/or elderly relatives	130	12.5%
Live with partner, children and elderly relatives	12	1.2%
Employment Status		
Full time employment	476	45.8%
Part time employment	191	18.4%
Student	63	6.1%
Unemployed and looking for work	171	16.4%
Full time carer/parent	139	13.4%

*Note. N* = 1040

### Analysis

The present study takes an exploratory approach and as such hypothesis testing using inferential statistics were not undertaken. Descriptive statistics were utilised to explore the data alongside a two-step cluster analysis. The two step cluster analysis was undertaken as it has been demonstrated to be useful in exploratory research to reveal natural but not obvious groupings in data (Walsh & Rísquez, 2020). Using the log likelihood method, the distance between continuous and categorical variables can be determined to reveal naturally occurring clusters (Walsh & Rísquez, 2020), in our case we were interested in naturally occurring demographic factors associated with covid compliance. A four-group model with a silhouette score of 0.8 was identified as the most optimal, with a Schwartz’s Bayesian Criterion (BIC) of 134.04. K-fold cross validation analyses were then utilised to assess the stability and generalisability of the clusters identified.

## Results

### Descriptive Statistics for Non-Compliance

Table 4 presents the overall participant responses to whether they broke UK Government social distancing laws by committing certain behaviours. The percentage column for each presents the overall percentage of respondents that said they committed each individual non-compliant behaviour.

**Table 4:** Self-Reported Public Compliance and Non-Compliance With Government Social Distancing Rules

Non-compliant behaviours with UK Government social distancing rules and guidance	*n*	%
Visited the shop not for essentials	281	27.02%
Met with friends or family not in household	202	19.42%
Stockpiled items	193	18.56%
Did not wash hands regularly	147	14.13%
Did not ensure I kept 2 meters apart from others	121	11.63%
Socialised inside a family or friend’s house	116	11.15%
Drove unnecessarily	113	10.87%
Exercised with someone not in household	92	8.85%
Hosted visitors inside own house	84	8.08%
Used public space not in accordance with rules	71	6.83%
Went out in public whilst suffering minor symptoms (did not self-isolate)	41	3.94%
Allowed children to socialise with their friends	31	2.98%^a^

^a^ only those who had children answered question, valid % (*n* = 703).

To establish the degree to which participants did not comply with UK Government Covid-19 rules, a total ‘non-compliance’ score was calculated for each participant across the 12 behaviours shown in Table 4. The average (mean) score for all participants was *M* = 1.42, *SD* = 1.83 suggesting that members of the public committed less than two behaviours which did not comply with Covid rules. However, the distribution is highly skewed. Further analyses highlighted that 39.6% of participants reported fully complying with Covid rules (i.e., did not engage in any of the 12 listed behaviours), 23.2% engaged in one behaviour, 17.5% engaged in two behaviours, 8.9% in three behaviours, 4.7% in four behaviours, 2.1% in five behaviours, 1.2% in six behaviours, 0.2% in seven behaviours, 0.5% in eight behaviours, 0.6% in nine behaviours, 0.8% in ten behaviours, 0.1% in eleven behaviours, and 0.1% in all twelve behaviours.

**Figure 1:**
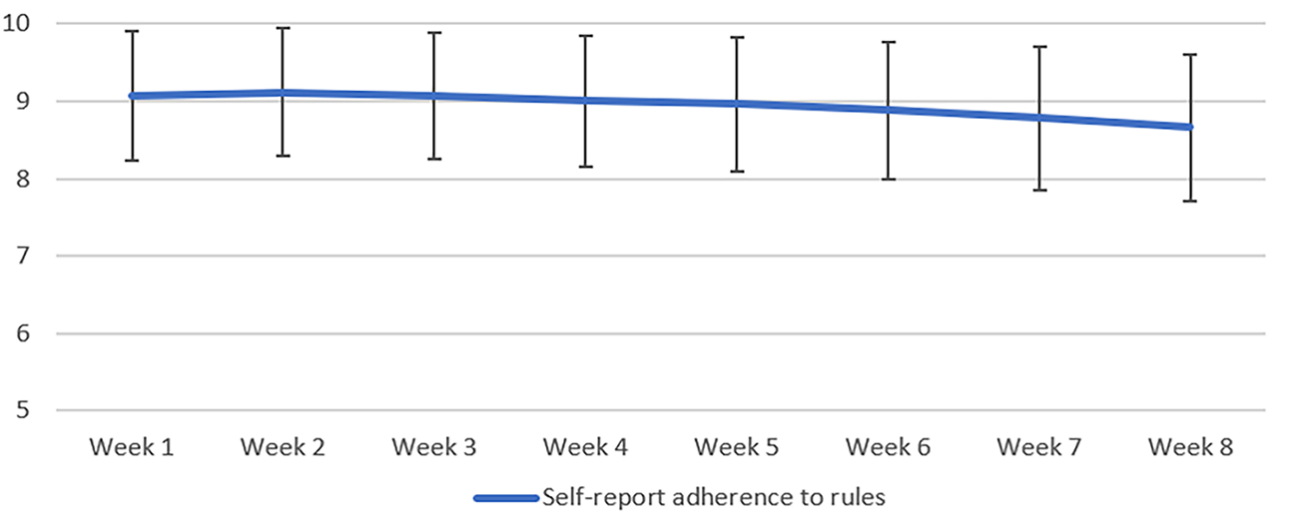
Self-Report Adherence to Rules

To examine the extent to which participants reported complying with Covid rules over time, all were asked to rate on a scale from zero to ten the adherence to the rules for the first eight weeks of lockdown. As can be seen in Figure 1, self-report adherence to guidelines and rules specified by the Government did not appear massively fluctuate over the initial eight weeks, however, it is the case that adherence is reported to gradually decrease from week two onwards. If taken over a longer period of say 12 months, then it is likely that this trend accelerated as compliance decayed.

### Two-Step Cluster Analysis

To understand the relationships between demographic variables, and the level of compliance a two-step cluster analysis was performed. Total violations were inputted as count data, and log-likelihood distance was utilised. The cluster analysis outlined six potential clusters with negative BIC values, however, the four-cluster model was optimal, as the ratio of BIC changes dropped from .232 to .040 at five clusters. The four-cluster silhouette measure of cohesion and separation score was .75 indicating good cluster quality. The four groups are displayed in Table 5 and demonstrate four clusters of the public. Interestingly Cluster 3 and 4 which represent 19% of the sample are responsible for nearly 60% of the total violations, with Cluster 4 being responsible for 14% of self-report violations when they only represent 2% of the sample. Table 5 outlines the demographic make-up of the four clusters.

**Table 5:** Cluster Analysis of Total Violations

Proportions/Means	Cluster 1 (Zero offending)	Cluster 2 (low-level breaches)	Cluster 3 (moderate breaches)	Cluster 4 (high level of breaches)
% of sample	39.6%	40.7%	16.9%	2.2%
% of breaches	0.00%	41.07%	44.60%	14.32%
Mean breaches	0.0	1.43	3.73	9.17
Mean age	48.67	47.75	43.14	33.50
Mean self-reported compliance	9.54	8.78	7.22	6.35
Largest gender proportion %	Female (54.9%)	Female (51.5%)	Male (54.5%)	Male (73.9%)
Parent	59.7%	57.0%	55.1%	78.3%
Largest ethnic group proportion	White or White British (82.3%)	White or White British (80.1%)	White or White British (79.0%)	White or White British (60.9%)
Second ethnic group	Asian or Asian British (9.2%)	Asian or Asian British 10.9%)	Asian or Asian British (10.2%)	Asian or Asian British (30.4%)
Religious	No (72.8%)	No (68.1%)	No (74.4%)	Yes (73.9%)
Monthly Income	£2,000 – £3,999 (39.1%)	£2,000 – £3,999 (36.4%)	£2,000 – £3,999 (39.2%)	£2,000 – £3,999 (34.8%)
Employment status	Full time employment (39.8%)	Full time employment (46.3%)	Full time employment (55.1%)	Full time employment (73.9%)
Furloughed	19.2%	18.4%	26.1%	52.2%
Key-worker	15.0%	20.6%	28.4%	47.8%
Location type	Suburban (46.6%)	Suburban (47.3%)	Suburban (43.2%)	Urban (69.6%)
Largest region	South-East (15.8%)	Greater London (15.1%)	Greater London (13.6%)	West Midlands (21.7%)
Living with	Live with partner (35.4%)	Live with partner (30.5%)	Live with partner and children (30.7%)	Live with partner and children (60.9%)
Family/friends suffered with Covid	12.9%	18.0%	15.3%	43.5%
Levels of trust in Government	49.5%	40.0%	38.1%	43.7%
Largest source of information	TV News Programmes (60.7%)	TV News Programmes (55.1%)	TV News Programmes (51.7%)	TV News Programmes (47.8%)

As can been seen in Table 5, the demographic profile of the cluster of individuals who did not breach the rules represent 39.6% of the sample. This group was older (*M*_age_ = 48.67), and predominantly female (54.9%) with most being White or White British (82.3%) and not religious (72.8%). Cluster 2 can be identified as the average rule breaker which accounted for 40.7% of the sample and accounted for 41.07% of breaches, with a mean of 1.43 breaches. This group tended to be older (*M*_age_ = 47.75), predominantly female (51.5%), or White British (80.1%) and not religious (68.1%). Cluster 3 are the above average rule breakers who represent 16.9% of the sample but are responsible for a disproportional number of breaches (44.60%). This group was younger (*M*_age_ = 43.14), predominantly male (54.5%), were White or White British (79.0%) and not religious (74.4%). Finally, as can be seen in Table 5 the prolific rule breakers were identified in Cluster 4 and accounted for 14.32% of breaches. This group was the youngest (*M*_age_ = 33.50), predominantly male (73.9%). It had a higher proportion of Asian or Asian British individuals (30.4%), had the highest percentage of key workers (47.8%) and the highest percentage of individuals who has been furloughed (58.2%) and was mostly religious (73.9%). Additionally, this group were far more likely to have had a friend or family member to have had COVID and was mostly comprised of individuals in families with young children. This group clearly stands out due to its high level of breaches and unique demographic profile.

To explore the specific breaches by cluster group, these are displayed in Table 6.

**Table 6:** Self-Report Specific Rule Breaking by Cluster Group

	Cluster 1		Cluster 2		Cluster 3		Cluster 4	
Rules	*N*	%	*N*	%	*N*	%	*N*	%
Met with friends or family not in household	0	0.0%	74	17.5%	105	61%	23	100%
Exercised with someone not in household	0	0.0%	23	5.5%	49	28.5%	20	87.0%
Used public space not in accordance with rules	0	0.0%	19	4.6%	32	18.4%	20	87.0%
Visited a shop unnecessarily	0	0.0%	140	33.1%	120	68.2%	21	91.3%
Drove unnecessarily	0	0.0%	48	9.5%	57	35.6%	18	78.3%
Socialised inside a family or friend’s house	0	0.0%	35	8.3%	59	33.7%	22	95.7%
Hosted visitors inside my own house	0	0.0%	5	1.2%	40	23.0%	20	87.0%
Allowed children to socialise with their friends	0	0.0%	3	1.0%	12	11.3%	16	76.2%
Stockpiled	0	0.0%	108	25.8%	68	39.3%	17	73.9%
Went out in public whilst suffering minor symptoms (did not self-isolate)	0	0.0%	6	1.6%	18	12.5%	17	73.9%
Did not keep 2 metres from others in public	0	0.0%	69	16.7%	43	24.6%	9	39.1%
I didn’t wash my hands regularly for 20 seconds using soap	0	0.0%	85	20.2%	54	31.0%	8	34.8%

**Figure 2:**
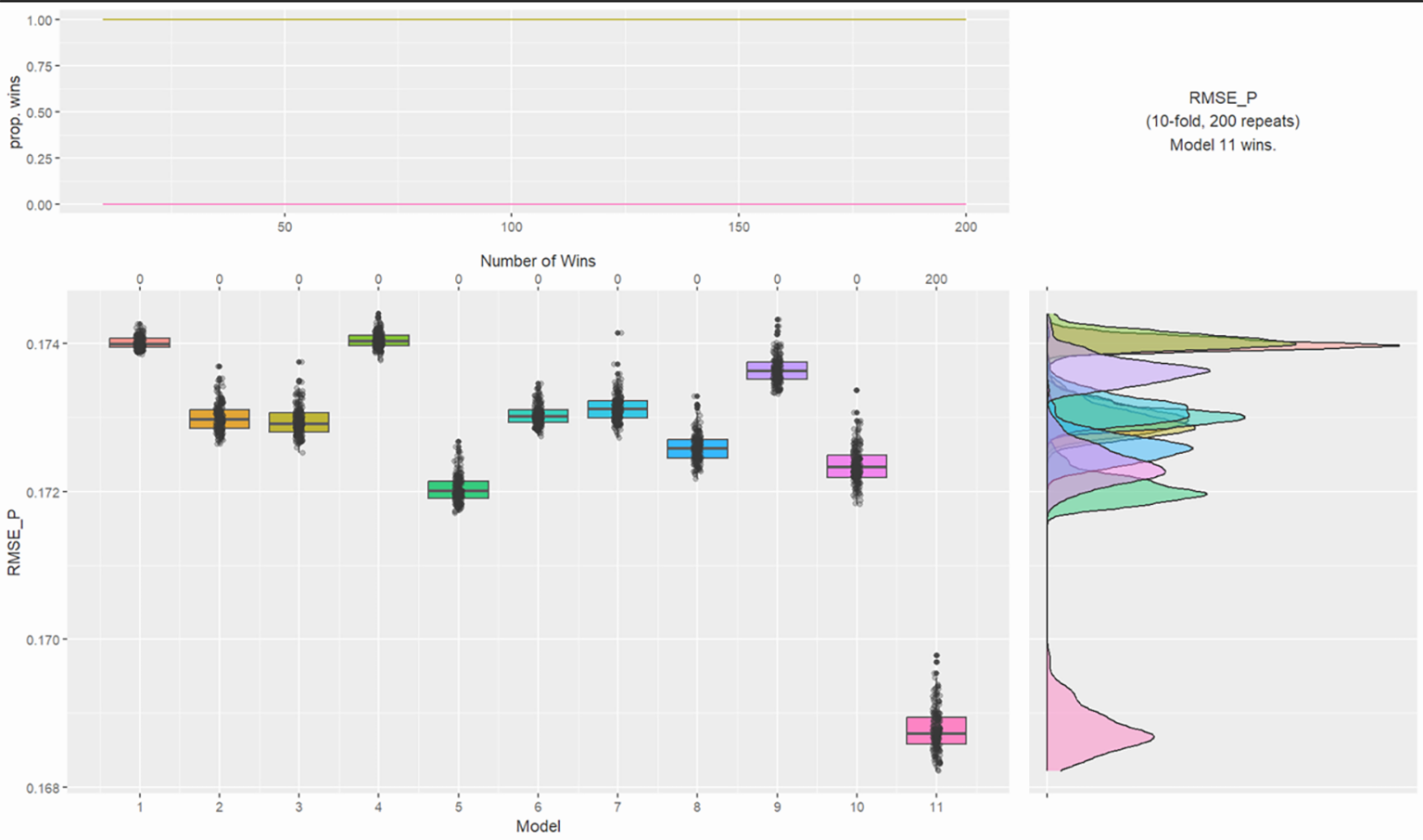
K-Fold Cross Validation Testing Cluster Group Performance for Whether Participants Were Warned by the Police for Breaching Covid Rules *Note*. Models: 1 = intercept, 2 = ethnicity and religion, 3 = age grouping and gender (interaction), 4 = parents, 5 = residential status and cohabitants, 6 = occupation and income, 7 = key worker, 8 = furloughed, 9 = previously had COVID, 10 = trust government and news source, 11 = 4 group cluster.

To understand the robustness of the four-model solution outlined in the cluster analysis, K-fold cross validation was undertaken (de Rooij & Weeda, 2020) to determine the impact of the clusters on whether the participants had been warned by the police for breaching covid rules. Alongside the four-cluster model, specific demographic variables, which have been associated with higher number of covid violations in existing literature were input as alternative models. Displayed in Figure 2 are the results for the K-fold cross validation. As can be seen the four-cluster model outperforms both the intercept and specific demographic variables with a Root Mean Square Error of Prediction (RMSE-P) of .169 versus .174, a difference of .005, suggesting that the specific cluster group that the participants belong to is the most important predictor of being spoken to by the police due to breaching covid rules. Of Cluster 1, 0.7% were warned by police; Cluster 2, 2.1%; Cluster 3, 6.3%; and Cluster 4, 52.2%.

**Figure 3:**
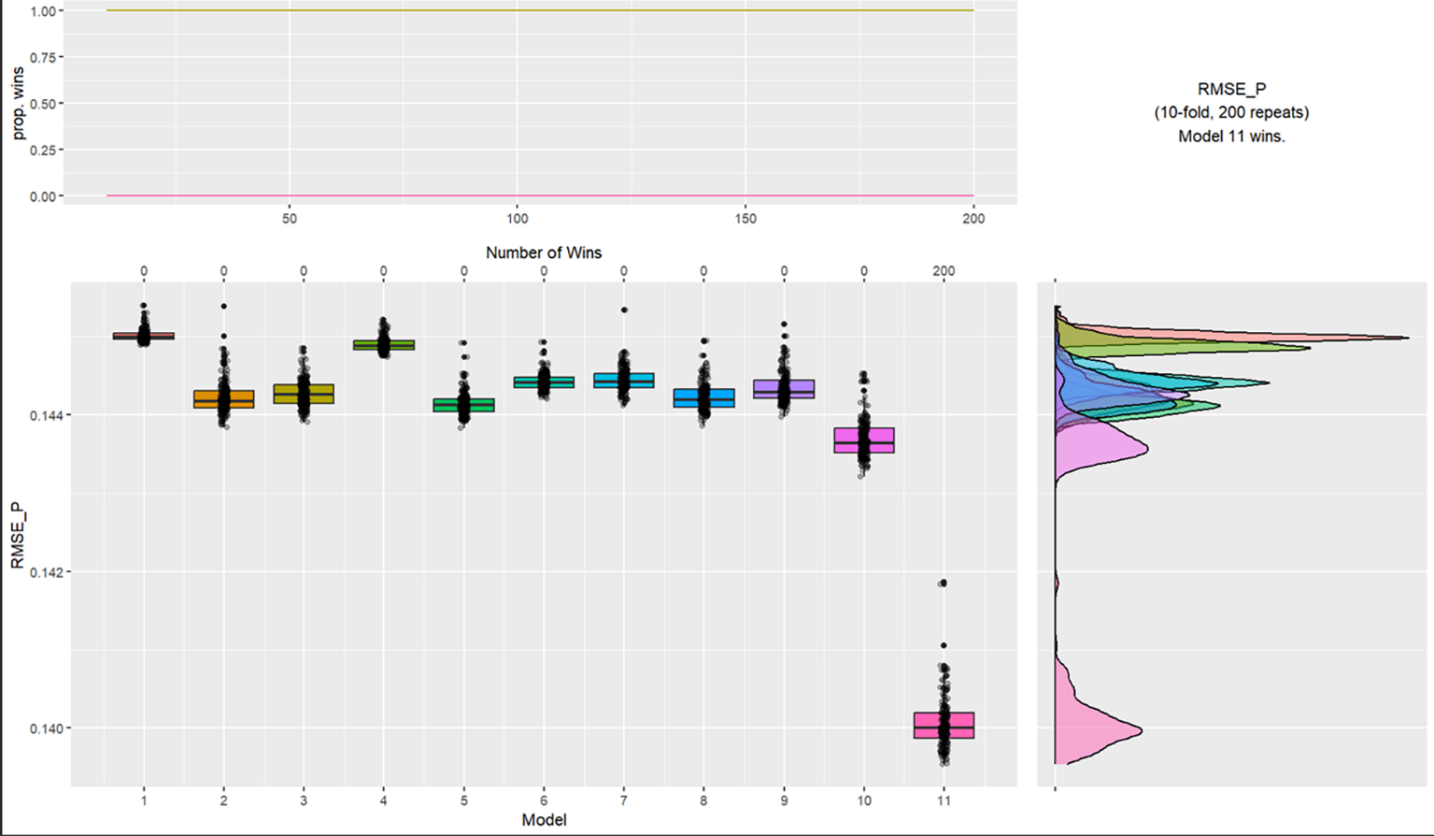
K-Fold Cross Validation Testing Cluster Group Performance for Whether Participants Were Arrested for Breaching Covid Rules *Note.* Models: 1 = intercept, 2 = ethnicity and religion, 3 = age grouping and gender (interaction), 4 = parents, 5 = residential status and cohabitants, 6 = occupation and income, 7 = key worker, 8 = furloughed, 9 = previously had COVID, 10 = trust government and news source, 11 = 4 group cluster.

A second K-fold cross validation (de Rooij & Weeda, 2020) was undertaken to assess the cluster performance in order to predict who was arrested for breaching COVD rules against the same demographic variables used in the previous validation. Again, the four-cluster model was superior as outlined in Figure 3 in predicting who was arrested with a Root Mean Square Error of Prediction (RMSE-P) of .145 versus .140, a difference of .005, suggesting that the specific cluster group that the participants belong to is the most important predictor of being arrested by the police due to breaching covid rules. Of Cluster 1, 0.0% were arrested or fined; Cluster 2, 1.7%; Cluster 3, 2.3%; and Cluster 4, 56.5%.

**Figure 4:**
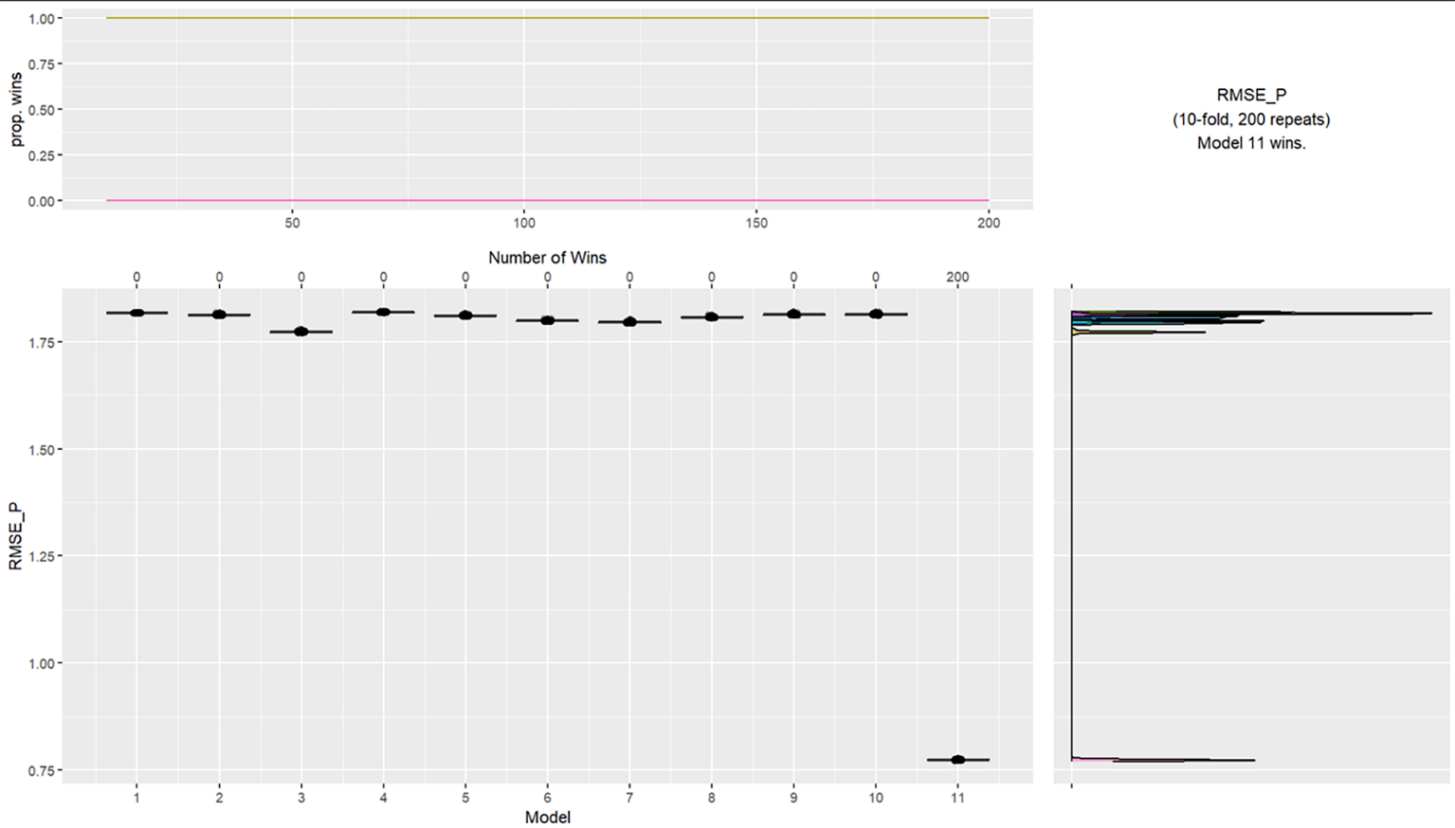
K-Fold Cross Validation Testing Cluster Group Performance for Whether Participants Were Arrested for Total Violations *Note.* Models: 1 = intercept, 2 = ethnicity and religion, 3 = age grouping and gender, 4 = parents, 5 = residential status and cohabitants, 6 = occupation and income, 7 = key worker, 8 = furloughed, 9 = previously had COVID, 10 = trust government and news source, 11 = 4 group cluster.

A third k-fold cross validation (de Rooij & Weeda, 2020) was undertaken to determine the cluster performance for predicating total number of violations. As would be expected, the four-cluster model was again far superior as outlined in Figure 4 with a Root Mean Square Error of Prediction (RMSE-P) of .773 versus 1.817, a difference of 1.044. The k-cross validations, provide support that the four-cluster model identified in the two-step cluster analysis is a robust predictor for those who breach COVID rules.

### Evaluating the Effectiveness of Messaging to Increase Compliance

**Figure 5:**
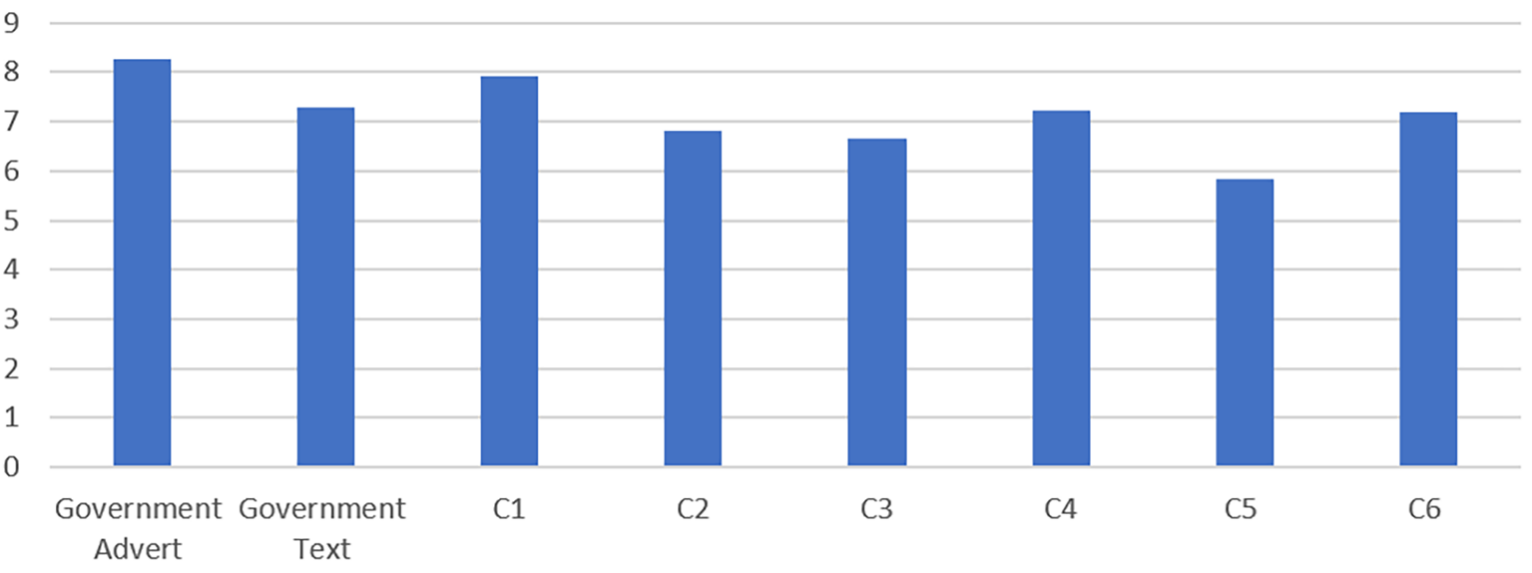
Participant Ratings of Messaging Effectiveness

The second exploratory objective of the present paper was to explore the effectiveness of the UK Government’s public messaging during the early stages of the pandemic, and to compare the degree to which participants felt these affected their compliance/non-compliance with some alternative messages that we developed, based upon focusing upon specific demographics. Figure 5 presents a comparison of the perceived effectiveness reported by participants for two messages used by the UK Government (in communications an advert and text message that were communicated at the start of the lockdown restriction) with six alternative communications. As can be seen in Figure 5, the initial Government advert was considered as the most influential by the participants, closely followed by Communication 1.

To examine whether the different messages were perceived to be influential according to the cluster in which participants were identified, the mean effectiveness scores based upon a 10-point scale of how effective participants considered the communications to be were analysed. As can be seen in Table 7, differences can be observed with regards to the effectiveness of each cluster groups rating. Generally, Cluster Groups 3 and 4 rated all communications as less effective than the other cluster groups, with Cluster Group 1 rating all communications as the most influential. This demonstrates that the communications were not necessarily ineffective as the Government advert was very effective for Cluster Group 1 who committed no violations. The communications were simply not as effective for the small sample of people in Cluster Group 3 and 4 who committed the most violations.

**Table 7:** Mean Effectiveness of Communications for Influencing Compliance per Cluster Group

	Cluster 1		Cluster 2		Cluster 3		Cluster 4	
Communication	*M*	*SD*	*M*	*SD*	*M*	*SD*	*M*	*SD*
Government Advert	8.9	1.7	8.3	2.0	7.0	2.5	6.7	3.5
Government Text	7.9	2.3	7.3	2.3	6.2	2.7	6.3	3.4
C1	8.4	2.0	7.9	2.2	6.8	2.7	7.2	3.2
C2	7.1	2.7	6.9	2.7	5.9	2.8	6.5	3.1
C3	7.2	2.9	6.5	3.0	5.5	3.2	6.4	3.0
C4	7.8	2.6	7.1	2.7	6.2	3.1	7.1	3.1
C5	6.4	3.3	5.7	3.2	4.7	3.3	6.6	3.4
C6	7.8	2.5	7.2	2.5	5.9	3.0	6.7	3.1
Mean Effectiveness	7.7	1.9	7.1	2.0	6.0	2.5	6.7	3.0

**Figure 6:**
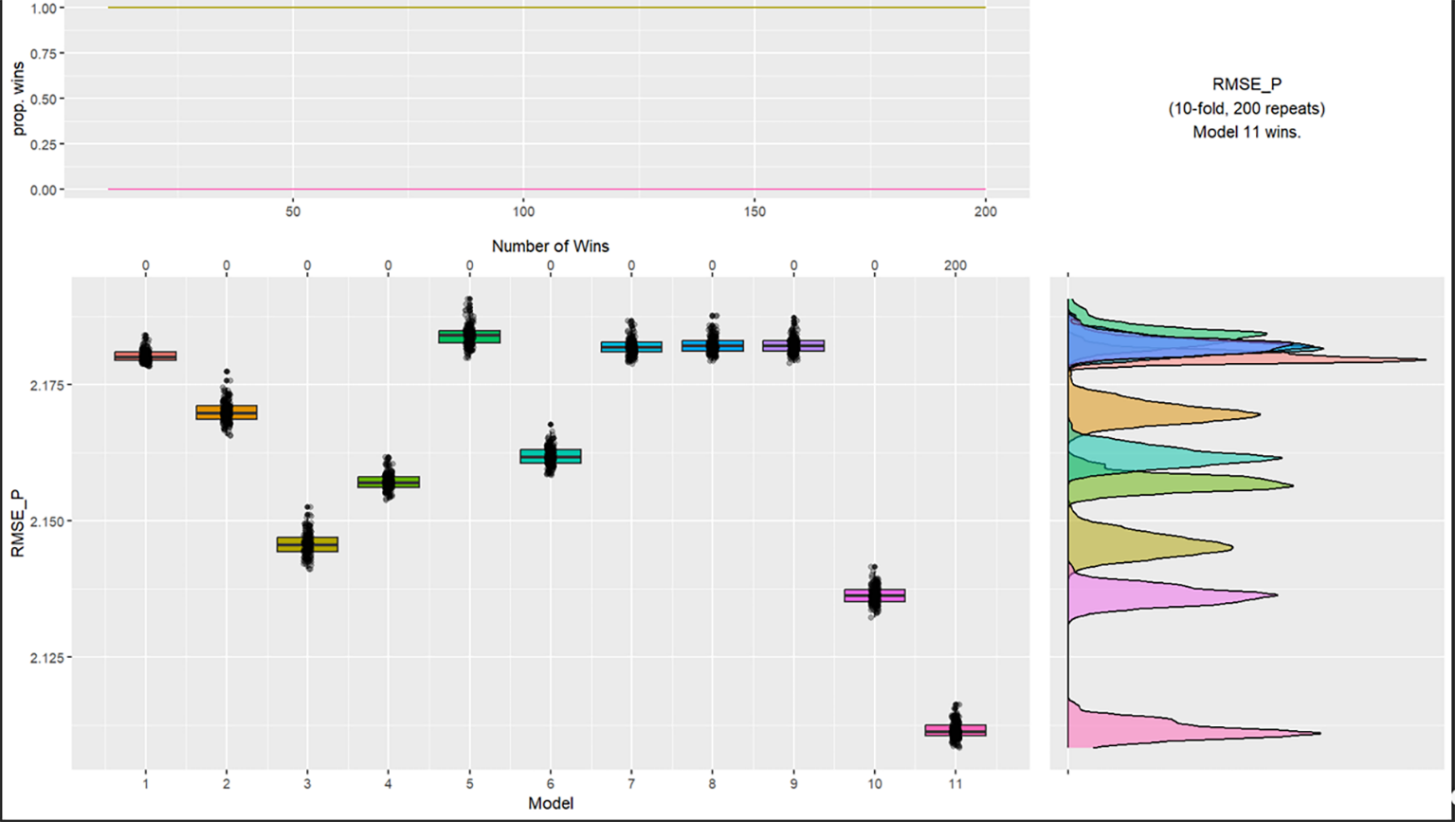
K-Cross Validation Analysis Effectiveness of Nudges *Note*. Models: 1 = intercept, 2 = ethnicity and religion, 3 = age grouping and gender, 4 = parents, 5 = residential status and cohabitants, 6 = occupation and income, 7 = key worker, 8 = furloughed, 9 = previously had COVID, 10 = trust government and news source, 11 = 4 group cluster.

K-fold cross validation was performed to determine the impact of the cluster groupings and alternative demographic variables on the mean effectiveness of all nudge interventions. The four cluster model was superior as outlined in Figure 6 to other models in the K-cross validation with a RMSE of 2.145, a mean difference of 0.035.

Finally, Table 8 demonstrated how best to influence the different cluster groups in terms the method of communication that was rated as the most effective and the messenger (e.g., who has sent it) that was rated as the most effective. For all groups the method of communications that was rated as the most influential was via TV advertisement sent by the NHS.

**Table 8:** Cluster Group Messenger and Mode of Communication Preferences

Communication Preferences	Cluster 1	Cluster 2	Cluster 3	Cluster 4	Total %
Method – Text	41.0%	43.1%	42.6%	43.5%	42.2%
Method – written letter	37.9%	35.1%	33.0%	13.0%	35.3%
Method – Facebook	13.1%	12.1%	17.0%	30.4%	13.7%
Method – Radio	19.2%	21.6%	20.5%	21.7%	20.4%
Method – TV advertisement	76.7%	71.6%	61.4%	52.2%	71.4%
Method – Games Platform Advertisement	1.0%	0.9%	3.4%	13.0%	1.6%
Method – Billboard	11.2%	15.6%	22.2%	26.1%	15.2%
Messenger – NHS	70.1%	71.6%	62.5%	60.9%	69.2%
Messenger – The Police	15.3%	17.0%	13.6%	26.1%	16.0%
Messenger – The Government	42.5%	37.8%	33.5%	21.7%	38.6%
Messenger – Local Council	3.4%	5.0%	7.4%	21.7%	5.1%
Messenger – Friends and Family	8.0%	11.6%	25.0%	43.5%	13.2%
Messenger – Scientific Opinion	60.7%	56.5%	58.0%	26.1%	57.7%

## Discussion

The present paper has endeavoured to shed-light on reasons for public compliance/non-compliance with social distancing rules introduced by the UK Government during the early period of the Covid-19 pandemic. The present study focused on self-reported non-compliance with UK Government rules and guidance’ by asking a representative group of UK participants about their partaking in specific social-distancing breaching behaviours.

Analyses revealed four clusters of UK residents emerged in regard to their Covid breaching behaviour. The first cluster represented those in society who did not breach any rules, who represented 39.6%. The second cluster represented the ‘average’ rule breaker who committed very few violations but non the less violated some of the COVID rules, but this was in line with the average individual, and they represented 40.7% of the sample. The third cluster represented those who engaged in several violations around two behaviours more than the average individual and this group represented 16.9% of the sample. The final cluster who only represented 2.2% of the sample habitually broke almost all rules and were responsible for 20% of the total violations. Combined, those identified in Cluster 4 and 3 represented only 19.1% of our sample but were responsible for 58.92% of total self-report violations. Indeed, this is interesting and provides crucial information as to who to target in future situations akin to the COVID 19 lock down.

Generally, our results demonstrate that the Governments messaging of “*If you can go out, you can spread it. People will die. Stay home. Protect the NHS. Save Live*s” was very effective in influencing compliance in those in Cluster 1 and 2 but was far less effective in Cluster Group 3 and 4. This suggests that such generic messaging was successful in eliciting a degree of conformity in these individuals, however for the prolific rule breakers identified in Cluster 4 and those committing more than the average in Cluster 3 these communications were not effective and suggests a more bespoke targeted approach is needed. That said our experimental communications did not perform better but these were created prior to revealing the clusters and future communications using the demographic data associated with our clusters may produce more effective communications that influence compliance in those who were identified in this study as responsible for many violations, whilst representing a small number of people.

Relatively few individuals reported adhering completely to all social distancing rules (< 40%) and that indeed, most reported regularly breaching the rules, with most reporting breaching at least at one rule during the first lockdown period. This extent of breaching the social distancing rules suggests that that there may have been issues with the communication of such rules. This is something that needs to be explored further, as alternative means and ways of conveying important ‘health-related messages’ may need to be found, and which are bespoke to different population demographics, if population behaviour change is to be increased for future pandemic scenarios. It could be argued of course that the lockdown rules were simply too novel and too restrictive as a package, for whole-scale public compliance to be a realistic prospect. Interestingly, scientists and health-care leaders stated early in the pandemic that younger people were less likely to experience ‘severe symptoms’, so not great leap of the imagination is necessary to explain why the younger participants reported being least compliant with the social distancing rules.

Based on our findings, a more effective communication strategy might have been to focus messaging more specifically: (1) upon the behaviours that would have the most success in reducing transmission such as self-isolation, and (2) using different messages and messengers for different population demographics. Different, for example, for communicating with young males as opposed to the whole population. However, this is an area for health scholars and policy makers. Furthermore, our prolific rule breakers represented a rather specific demographic background, and this should be noted for future pandemics. In particular, the fact that this group consisted of mostly religious individuals and a high proportion were either key workers or had been furloughed from their job.

Arguably, the least surprising finding of present paper as with previous research (e.g., Smith et al., 2020) was that it was young male participants that reported most non-compliance with social-distancing rules. As alluded to, this presents opportunities for targeting this demographic in the future, that said the results of the latter part of this study indicate that males in general may be more difficult to influence to influence using communications. Other influential factors included ‘identifying as a key worker’ and the perception of neighbours breaching the rules. Again, this is like (Smith et al., 2020) and provides the suggestion for the future that effective communications may seek to reduce cognitive biases and dissonance in this area. For example, ‘*everyone else is doing it so it is ok If I do’,’ it is okay because I am a key worker’*. Seeking to target and ‘neutralise’/reduce such justifications in decision-making may be an important way to influence the behaviour of these groups. This may also be true for other over reported demographics in the fourth cluster group, for example young families with young children and those members of the public who had friends who had suffered with COVID.

Research has consistently found that behavioural change weans (or decays) over time (e.g., Wu et al., 2022) and our findings here are no different with more frequent breaches of the rules being reported over time. This is perhaps best explained in terms of: (1) growing public intolerance with kerbs on freedoms, (2) the roll-out of the Covid-19 vaccination programme, and (3) a large percentage of the population having had the virus: (a) only experienced ‘mild’ symptoms, and (b) scientific knowledge that having had the virus is the best form of protection against severe symptoms in the future. Additionally, the four distinct clusters represent those at the start of the pandemic and not as it went on, it is likely that as the pandemic progressed the distinct groups of rule breakers would look very different.

The present paper provides an estimation of whether official warnings and contact with the police (due to breaches in social distancing) positively affect future behaviour. To the authors’ knowledge this has not been studied, and the paper demonstrates an estimation that only 2% of the population were issued with fines and 3% of the population issued with warnings. As such despite rather high levels of self-report breaches in the rules only a fraction of individuals interacted with the police due to their behaviour. Those who were, tended to be those identified as the prolific rule breakers.

This study contributes to furthering our understanding about human behaviour during the pandemic and provides some specific detail as to the types of behaviour that were undertake in contradiction to the rules and those likely to breach rules more than others. The paper also provides some understanding about the effectiveness of communications during the pandemic and as discussed throughout the governmental communication that read “*If you can go out, you can spread it. People will die. Stay home. Protect the NHS. Save Lives”* was more effective than any of the communications designed in this study for targeting specific demographics. In the absence of the knowledge of who was breaching the rules and focusing on the whole population this demonstrates that the governmental communication was effective on a population level. Had the government had the insights of the present data to design the communication further, greater levels of behavioural change may have been achieved. This research also demonstrates that the most influential messenger would be the NHS and the platform for communicating that would be the most effective according to the present sample is TV advertisements.

### Theoretical Contribution

The present paper did not aim to be an in-depth evaluation of the application of nudges. However, there are important theoretical contributions to consider. The findings support the idea that nudge approaches are only effective when the demographic is known; none of the bespoke nudges developed in this study performed better than the generic nudges created by the UK Government. Furthermore, the concepts of messenger and mode of communication, which are purported to be essential in nudge design, have demonstrated their importance. The findings reported here highlight differences in participants' perceptions of influence regarding who and how compliance can be elicited.

### Policy Implications

The findings reported here provide important insights regarding the behaviour of the UK public during the pandemic and potential approaches to achieving greater levels of behavioural change in future situations where we may need to adjust the behaviour of the population. The lessons learned from the pandemic based upon the present data, which may be of interest to policy makers are as follows:

- We are human and not social distancers **—** the public struggle to implement such mass change to their behaviour for prolonged periods.
- Focusing communications surrounding specific behaviours that may have the most effect at reducing transmission may prove to be more effective.
- Addressing cognitive biases which allow neutralisations for offending to take place may prove fruitful in reducing breaches in rules.
- Utilising NHS (medical) employees via TV advertisements may have the most powerful effect in accomplishing behavioural change.
- A small proportion of individuals are responsible for undertaking most behaviours against the rules, influencing these demographics would have the greatest overall effect.

Our take home suggestion for Government officials is not so much to challenge the initial use of message, but with the lack of understanding that it’s efficacy in terms of behaviour change was always likely to reduce/decay, with time. Based on the findings of the present research, in our opinion, to better sustain public compliance then subsequent/ follow-up messages need to: (1) target different population demographics, and (2) target specific behaviours for compliance, such as ‘do not mix with other households’ rather than continuing to refer to social distancing rules as one homogenous package.

### Limitations of the Study

The present study was undertaken at a time when the UK was facing an unprecedented public emergency, and the UK public was entering an unknown period of change and uncertainty. The findings provide important insights and information to policy makers and those tasked at implementing behavioural change if it is needed in the future. However, it does have several limitations that must be acknowledged. The study utilised a self-report sample which may not represent the true extent of breaches in social distancing due to socially desirable responding and is only representative to those who use the internet. Additionally, the paper cannot provide a full understanding of how the government’s communications were designed and by no means includes all the messages that were communicated but simply two examples. That said, the evidence provided here demonstrates the communications were more effective than alternatives developed in the present study. This might of course simply have been due to a ‘primacy effect’ with ‘*Stay home. Protect the NHS. Save Lives’*, being a clear and unequivocal first message for a surprised and disorientated UK public.

### Directions for Future Research

The present paper provided exploratory insights, highlighting numerous areas for future exploration. Due to the cross-sectional nature of this research, future studies should adopt a longitudinal design to better understand how behaviour changes over time and whether different clusters of individuals engage in rule-breaking at various time points. Additionally, the paper did not address why certain demographics may engage in rule-breaking, so future research directly addressing this issue would be valuable. The paper also tested nudges prior to understanding the demographics of rule-breakers; thus, future research should explore whether the data outlined here can be effectively harnessed to design influential nudges to improve compliance. Finally, future research should aim to corroborate or refute our findings using alternative data sources, rather than relying solely on self-report data. Studies utilising police data or data collected by local councils regarding rule-breakers would provide alternative insights that may not suffer from the biases inherent in self-reporting.

We acknowledge that, as hindsight is the only exact science, the pandemic provided a unique opportunity to examine public compliance and behavioural change. The insights gained from the four clusters of pandemic rule breakers identified in this study provide valuable knowledge that can better prepare us for future pandemic scenarios. Furthermore, the present study provided a first insight into the effectiveness of communications that were used for influencing compliance.
